# A Simple Pluronic F-127 Pretreatment Enables Near-Complete Recovery of *C. elegans* from Plastic Pipette Tips and Microcentrifuge Tubes

**DOI:** 10.17912/micropub.biology.002332

**Published:** 2026-10-22

**Authors:** Crystal A. Shults, Jacob Manjarrez

**Affiliations:** 1 Biochemistry and Microbiology, Oklahoma State University Center for Health Sciences, Tulsa, OK, United States

## Abstract

Adhesion of *Caenorhabditis elegans* to plastic pipette tips and microcentrifuge tubes is a common source of sample loss and variability in experimental workflows. Here, we describe a simple consumable-preparation method using 0.5% Pluronic F-127 to reduce worm sticking and improve recovery from plastic surfaces. As a less hazardous alternative to traditional siliconization procedures, Pluronic-treated tips and tubes enabled consistent worm release, while increasing the concentration to 1% provided no additional benefit. Recovery efficiency was not retained following autoclaving of treated consumables. This protocol offers a practical and accessible approach to improving reproducibility in *C. elegans* handling and sample-processing workflows.

**Figure 1. Evaluation of Pluronic F-127 treatment for improving worm recovery from plastic consumables:** (A) Recovery of worms using untreated and Pluronic F-127-treated consumables at a final concentration of 0.5%. Treatments are designated as follows: Rtu, untreated tube; Rtip, untreated tip; Ptu, Pluronic F-127-treated tube; and Pip, Pluronic F-127-treated tip. Pluronic F-127-treated tubes and tips consistently exhibited higher recovery rates than untreated consumables, indicating reduced worm retention when using standard plastic laboratory materials. (B) Recovery of worms using untreated and Pluronic F-127-treated consumables at a final concentration of 1.0%. Percent recovery was lower than that observed with the 0.5% Pluronic F-127 treatment, indicating increased worm retention compared with the 0.5% treatment condition. (C) Recovery of worms using 0.5% Pluronic F-127-treated consumables with post-treatment autoclaving. Recovery rates were lower than for non-autoclaved Pluronic F-127-treated tubes and tips, suggesting that autoclaving reduces treatment effectiveness. (D) Recovery of worms using untreated and Pluronic F-127-treated consumables at a final concentration of 0.5% with 200 µL tips. There was significance different in recovery from the total to the plate Plots in panels A-D were generated and analyzed using GraphPad Prism v11.0.2, paired t-tests with p-values of * (0.05), ** (0.01), *** (0.001), and **** (0.0001). Error bars indicate SEM for each category, from at least three independent replicates, with an average of 30-40 per experimental trial; data are presented with all totals normalized to 30 total worms/replicate.

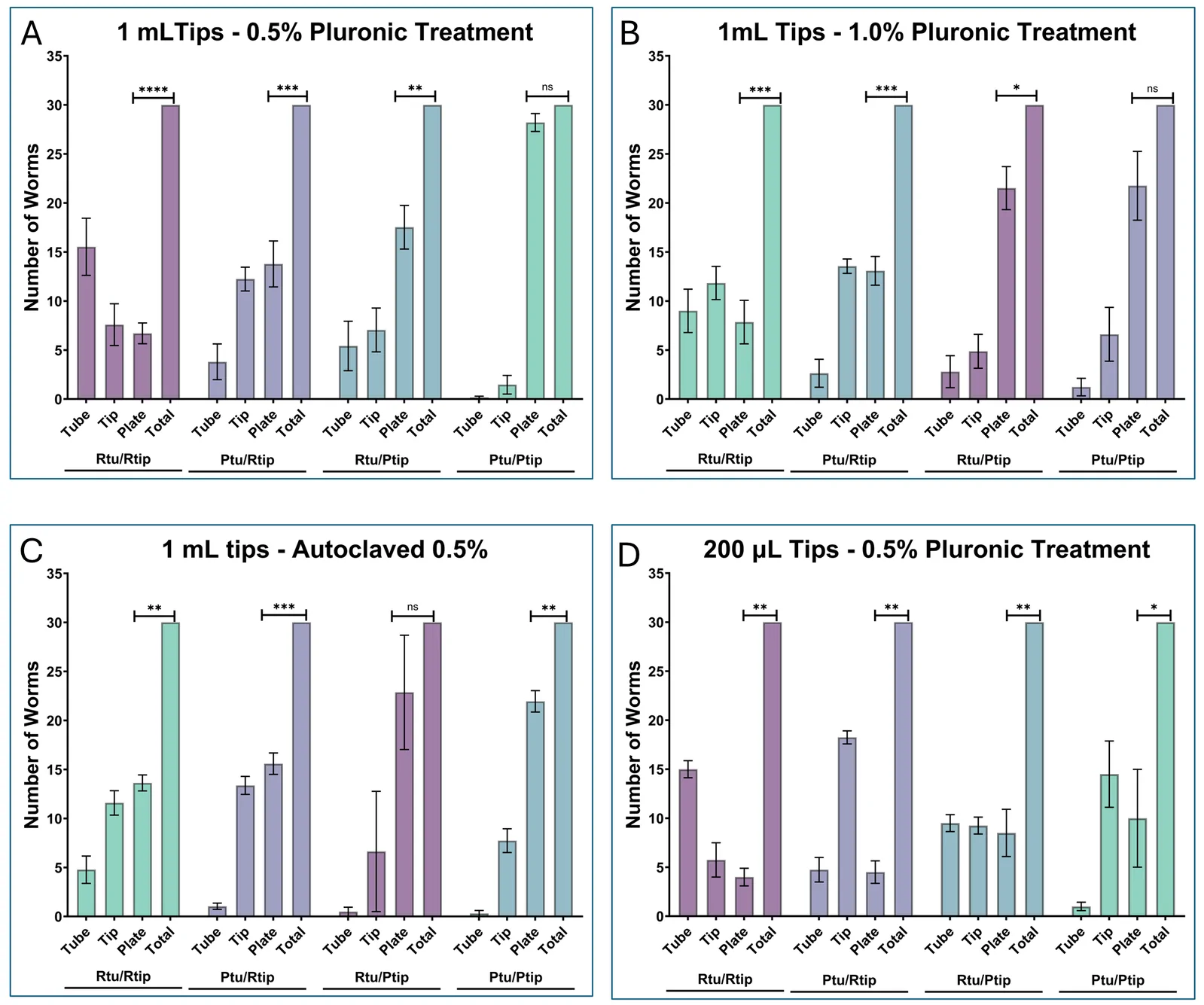



## Description

The issue of nematodes sticking to plastic pipette tips and sample tubes has been an obstacle to the accurate transfer of animal samples, indicating that worm adhesion to plastic is a common, reproducibility-limiting challenge in *C. elegans* workflows. Glass pipettes are repeatedly recommended as a corrective measure, with several sources directly stating that worms stick to plastic and that glass pipettes reduce loss due to adhesion (Rivera et al. 2022; Wilson, Qadota, and Benian 2012; Cao et al. 2023; Scanlan et al. 2018). There are some practical strategies outlined to counteract the sticking. A consistent, applicable approach is to include detergents in wash or resuspension buffers to minimize sticking; this includes Tween 20 (0.1%) or Triton X-100 (0.1%) in conjunction with M9 or PBS-T to reduce adhesion to surfaces (Rivera et al. 2022; Sin et al. 2018; Wilson, Qadota, and Benian 2012; Cao et al. 2023; Haroon and Vermulst 2019; Shaham 2006). In addition to detergent-based surfaces, practical adjustments such as pipette tip priming, gravity settling, and detergent-enabled resuspension are described to improve recovery and reproducibility across protocols (Rivera et al. 2022; Cao et al. 2023; Scanlan et al. 2018). This emphasizes the importance of minimizing surface adhesion for accurate handling of worms in FISH, immunostaining, RNA/protein assays, and high-throughput workflows, where glass versus plastic tools are recommended as a best practice (Rivera et al. 2022; Wilson, Qadota, and Benian 2012; Cao et al. 2023; Scanlan et al. 2018). Some protocols use complementary steps combining the use of M9 buffer with Tween 20/Triton X-100, specific centrifugation speeds, and careful transfer practices to minimize worm loss due to sticking during sample processing (Rivera et al. 2022; Sin et al. 2018; Scanlan et al. 2018).

We found that prewashing and drying pipette tips and microcentrifuge tubes with 0.5% Pluronic F-127 (Thermo Fisher Scientific Chemicals 2011) provides an effective surface treatment for future use. This approach offers an alternative to the siliconization method described in *WormBook: Methods in Cell Biology* (Shaham 2006), in which tubes are coated by dipping them in 2% dimethyldichlorosilane (Fisher Scientific Company 2010) dissolved in 1,1,1-trichloroethane (Lab Alley 2018)and then dried to reduce adhesion. Similar anti-sticking effects can be achieved using Pluronic F-127 without the need for these more volatile reagents.

Pluronic F-127 is used at 5% for coating microfluidic tubing and devices (NemaLife); however, we show that a concentration of 0.5% is sufficient to achieve consistent release of worms from plastic pipette tips and microcentrifuge tubes; SD/SEM 2.234/0.912 ( Figure 1A ), as this concentration has reduced variation. Increasing the concentration to 1% did not further improve recovery; SD/SEM = 7.838/3.505 ( Figure 1B ). Particularly important, treated tips did not retain their enhanced recovery efficiency following autoclaving, nor does the treatment show effectiveness for 200 µL tapered or wide bore tips ( Figure 1C- D; Sup. A). There are some indications that autoclaved tubes treated with 0.5% Pluronic F-127 do retain enhanced recovery; however, this does show specificity for 1 mL tips, as use with the 200 µL tips shows impaired recovery even without being autoclaved. A graphical overview of the protocol is provided as Supplement B, and the complete procedure is described in the Methods section to facilitate adoption by both visual and text-based learners.

## Methods

**Culturing and collecting worms**

**Culture** *C. elegans* , strain N2 , at 20°C on nematode growth medium (NGM) agar plates. Plates were seeded with E. coli strain OP50 and grown overnight at 20°C. Age-synchronize *C. elegans* N2 and grow on NGM/ OP50 plates incubated at 20°C until worms reached the L4 stage.

**200 – 1000 µl Pipette Tips and 1.5ml Tube Pluronic F-127 preparation**

(1) Set up three 50ml conical tubes, 40mls each. First conical tube: 70% ethanol wash. Second conical tube: tested % of Pluronic F-127. Third conical tube: Tris-HCl (100mM, pH 8.0) buffer.

(2) For tip Pluronic F-127 preparation, set the pipette to the full volume of the tip.

(3) Pipette the full volume of the tip up and down 10 times in the first conical (70% ETOH). If washing many tips, replace 70% ETOH as needed.

(4) With the same empty tip, pipette the full volume up and down 10 times in the second conical (% of Pluronic F-127 being tested). If washing many tips, replace % Pluronic F-127 as needed.

(5) With the same empty tip, pipette the full volume up and down 10 times in the third conical (Tris-HCl buffer). If washing many tips, replace Tris-HCl buffer as needed.

NOTE: If you do not use the tips immediately, store the treated tips at 4°C until needed for the experiment. Warm to room temperature before use. Ensure they are fully at room temperature before use.

(6) For 1.5ml microcentrifuge tube Pluronic F-127 preparation, use the same conical tube setup as in step 1.

(7) Pipet 1.5ml of 70% ETOH into each microcentrifuge tube. Place the tubes on a tube rotator at room temperature for 5 to 10 minutes at 25 rpm. Empty tubes.

(8) Pipet 1.5ml of the % Pluronic F-127 solution into each tube. Set the tubes on the rotator at room temperature for 15 minutes at 25 rpm. Empty the tubes.

(9) Using one pipette tip, pipette 1.5mL of Tris-HCl buffer into each tube three times to rinse. Do not change the tip for each tube. Fill and remove Tris-HCl using the same tip. Remove as much of the Tris-HCl buffer as possible from each tube. Blot upside down on a paper towel and place on the biosafety hood edge for 15 minutes.

NOTE: If not using the tubes immediately, store the treated tubes at 4°C until needed for the experiment. Warm to room temperature before use. Ensure they are fully at room temperature before use.

**0.5% and 1% Pluronic F-127 experiments**

(1) Prepare tips and tubes with Pluronic F-127.

(2) Put 500 µl M9 into each tube (treated tubes: 0.5% or 1% Pluronic F-127, and untreated tubes).

(3) Pick worms (strain N2 )(stage L4) and place them in each tube directly from the pick (swishing the pick a minimum of 10 times in the M9).

(4) Incubate at room temperature for 10 minutes for worms to settle. Centrifuge for 2 minutes at 1500 rpm. Check for floating worms. Draw off M9 buffer to the 100 µL mark on the tube carefully, not to disturb the worm pellet.

**NOTE** : Untreated tubes may not pellet worms completely. If necessary, centrifuge untreated samples a second time before removing the supernatant.

(5) Pipet up and down, with treated or not treated tips, three times, then transfer to an unseeded NGM plate for counting.

(6) Check and count the tube and tip under a microscope for leftover worms. Count worms on the NGM plate.

**Autoclave-treated (0.5% Pluronic) tips and tubes experiment**

(1) Autoclave-treat tips and tubes (0.5% Pluronic F-127), gravity cycle, 121 degrees C, 20 min sterilization, 5 min dry cycle. Cool the tips and tubes to room temperature before use.

(2). Put 500 µl M9 into each tube (treated tubes: 0.5% or 1% Pluronic F-127, and untreated tubes).

(3) Pick worms (strain N2 , L4) and place them in each tube directly from the pick (swishing the pick a minimum of 10 times in the M9).

(4) Incubate at room temperature for 10 minutes for worms to settle. Centrifuge for 2 minutes at 1500 rpm. Check for floating worms. Draw off M9 buffer to the 100 µL mark on the tube carefully, not to disturb the worm pellet.

**NOTE** : Untreated tubes may not pellet worms completely. If necessary, centrifuge untreated samples a second time before removing the supernatant.

(5) Pipet up and down, with treated or not treated tips according to the experimental group, three times, then transfer to an unseeded NGM plate for counting.

(6) Check and count the tube and tip under the microscope for leftover worms. Count worms on the NGM plate.

## Reagents

Ethanol 200 proof ACS/USP grade (Pharmco)

Pluronic F-127 (Sigma-Aldrich, cat no. P2443)

Water, deionized, ultra-filtered, autoclaved (Fisher Chemical, cat. no. W2-20)

Sodium Chloride (Fisher Chemical, cat. No. S271)

Agar (Apex, cat. no. 20-273)

Peptone (Fisher Bioreagents, cat. no. BP1420)

Cholesterol (MP Biomedicals, cat. no. 02101380)

Potassium dihydrogen phosphate (Fisher Chemical, cat. no. P285)

Potassium phosphate dibasic (Fisher Bioreagent, cat. no. BP363)

Magnesium sulfate (Fisher Chemical, cat. no. M63)

Calcium chloride (Spectrum, cat. no. C1096)

**NGM culture plates**

0.25% Peptone,0.3% sodium chloride,2% agar, 1mM calcium chloride, 1mM magnesium sulfate, 25mM potassium phosphate buffer (pH 6), and 5µg/ml cholesterol.

**Buffers**

Tris-HCl 1M Solution, pH 8.0 (Thermo Scientific, cat. no. J22638-K2): diluted with sterile water to 100mM and sterilized by .22µm filter.

M9 buffer: 22mM KH _2_ PO _4_ , 42mM Na _2_ HPO _4_ , 86mM NaCl, 1mM MgSO _4_ ; sterilize by .22µm filter.

## Data Availability

Description: (A) Shows recovery of worms using untreated and Pluronic F-127-treated consumables at a final concentration of 0.5% with 200 µL wide-bore tips. There was significance different in recovery from the total to the plate; for this experiment, only two independent replicates were obtained for the untreated tips and tube condition. (B) Schematic overview of the protocol used to prepare Pluronic F-127-treated tubes and pipette tips; the figure was created using BioRender.com. Resource Type: Workflow. DOI: https://doi.org/10.22002/79eap-zpf44
